# A Virtual Reality–Supported Intervention for Pulmonary Rehabilitation of Patients With Chronic Obstructive Pulmonary Disease: Mixed Methods Study

**DOI:** 10.2196/14178

**Published:** 2020-07-07

**Authors:** Timothy Jung, Natasha Moorhouse, Xin Shi, Muhammad Farhan Amin

**Affiliations:** 1 Manchester Metropolitan University Business School Manchester United Kingdom; 2 Burnett Edgar Medical Centre Central Drive Walney United Kingdom

**Keywords:** virtual reality, COPD, rehabilitation

## Abstract

**Background:**

The uptake of traditional pulmonary rehabilitation classes by patients with chronic obstructive pulmonary disease (COPD) is poor because of personal factors that prevent accessibility to the venue. Therefore, there is a need for innovative methods of pulmonary rehabilitation, and virtual reality (VR) could be a promising technology for patients with COPD to access services remotely.

**Objective:**

This study aimed to investigate whether VR improves compliance with pulmonary rehabilitation among patients with COPD, a particularly vulnerable patient group (Medical Research Council [MRC] 4 or 5), and whether VR provides a credible alternative to traditional pulmonary rehabilitation programs.

**Methods:**

This was an 8-week patient trial using an innovative VR pulmonary rehabilitation program. A purposive sample of 10 patients with COPD graded MRC 4 or 5 and registered at a selected health care center and a hospital in Cumbria, United Kingdom, were included. Qualitative (focus groups and interviews) data were collected, and to further support the qualitative findings, quantitative data (self-report patient surveys) were gathered before and after the 8-week trial. The 5 self-reported surveys included the Patient Activation Measure, Generalized Anxiety Disorder-7, Patient Health Questionnaire-9, Short Physical Performance Battery, and the Edmonton Frail Scale.

**Results:**

In a thematic analysis of the qualitative data, 11 themes emerged specific to delivering pulmonary rehabilitation using VR. The quantitative data further support the qualitative findings by revealing significant improvements in all physical measures.

**Conclusions:**

Overall, this study demonstrates how remotely supervised VR-based pulmonary rehabilitation could help to overcome current issues and limitations associated with providing this service to patients with COPD at scale.

## Introduction

### Background

Pulmonary rehabilitation is a nonpharmacological intervention designed for patients with chronic obstructive pulmonary disease (COPD), involving supervised exercise training, disease education, and behavioral interventions [1]. It is now one of the most effective treatments for significantly improving symptoms of dyspnea (ie, breathlessness) [2-4], exercise capacity, and improved quality of life [2,4-6] and reducing anxiety and depression in patients with COPD [1,7]. However, the uptake of pulmonary rehabilitation is poor, and completion rates are low [1,7,8]. Patients who qualify for pulmonary rehabilitation are those graded 3 or above on the Medical Research Council (MRC) breathlessness scale, which is a validated measure of disease severity used in many scientific studies across the world, given that this particular group is at high risk of exacerbation and hospital admission [9-11]. However, patients have difficulty attending classes because of lack of transport and geographic distance to a program, fatigue, lack of motivation, inconvenience, disruption caused to daily activities, and the quality of conversation that health care professionals have with patients about pulmonary rehabilitation [12,13]. Depression, burden of illness, low awareness of rehabilitation, and knowledge of and disbelief in beneficial rehabilitation outcomes are also considered barriers to patient uptake [14-16]. Therefore, researchers and practitioners are searching for innovative methods to deliver more engaging rehabilitation for patients with a variety of long-term conditions that affect physical activity.

More recently, there has been an increase in research focusing on the effects of virtual reality (VR) for patients with chronic diseases that inhibit physical activity. For example, studies focusing on individuals with Parkinson disease have investigated the effects of gait training with VR [17], whereas others have compared home-based VR balance training with conventional home-based balance training [18]. The physical and psychological benefits of VR for patients who have had a stroke have also been explored [19,20]. However, despite the extant research in this area, studies investigating VR for physical training rehabilitation programs have demonstrated varying results, which means that researchers and practitioners are unsure about its true impact [21,22]. The need for innovative methods of pulmonary rehabilitation is evident, and VR could be a promising technology for providing a convenient and remotely accessible pulmonary rehabilitation program to patients with COPD. COPD places a significant financial burden on individuals and societies worldwide and is a global public health burden that needs to be addressed to reduce the substantial cost associated with the disease and improve patients’ quality of life [23,24]. Remotely supervised VR could complement conventional therapy [25], which has demonstrated poor uptake because of patients’ personal barriers [8]. However, to the best of our knowledge, limited research has investigated the benefits and outcomes of VR for pulmonary rehabilitation, which leaves an important area of research requiring further investigation. To address this gap, this study aimed to investigate whether VR improves the compliance of patients with COPD for pulmonary rehabilitation, with a particular focus on the vulnerable patient group (MRC 4 or 5), and whether this technology provides a credible alternative to traditional pulmonary rehabilitation programs. The results of this study provide significant theoretical contributions to the field of health care research and provide important implications for health practitioners.

### What Is Already Known on This Topic?

- COPD is a global public health burden that needs to be addressed to reduce the substantial cost associated with the disease and improve the patient’s quality of life.
- Remotely supervised VR could complement conventional therapy, which has demonstrated poor uptake due to patients’ personal barriers.
- Limited research has investigated the benefits and outcomes of VR for pulmonary rehabilitation, which leaves an important area of research requiring further investigation.

### What This Study Adds?

- VR provides the opportunity for an interactive and visually stimulating approach for use in clinical treatment and could provide pulmonary rehabilitation to patients with COPD who cannot easily access traditional rehabilitation methods.
- This study provides a significant contribution to health care research investigating the benefits of implementing digital technologies to improve rehabilitation, with particular contribution to pulmonary rehabilitation research.
- Important practical implications for the health care industry and medical practitioners on the benefits of VR for pulmonary rehabilitation are offered.

### Literature Review

#### Technology-Facilitated Methods for Pulmonary Rehabilitation

COPD is characterized by progressive airflow obstruction and cannot be fully reversed [26]. Patients with COPD find it difficult to engage in physical activity and often have episodes of exacerbation, including breathlessness and coughing, which provoke anxiety and episodes of panic leading to an overall decline in everyday function and overall quality of life [26-29]. The psychological consequences of COPD include symptoms of depression; general anxiety [30-35]; and psychological repercussions, including fear, panic, loss of confidence, and social isolation [36,37], which have important implications for the adoption and maintenance of healthy behaviors [38]. In turn, this restricts the patients’ ability to perform daily activities, and owing to the fear of dyspnea, they often avoid physical activity and social events, which may contribute to an increased likelihood of depression in elderly patients with COPD [30,36,37]. In addition, an increasingly sedentary lifestyle leads to muscle weakness, further reductions in physical activity, reduced exercise capacity, and even more severe symptoms [39,40]. The MRC breathlessness scale measures perceived respiratory disability and is widely used to describe patient cohorts and stratify them for interventions such as pulmonary rehabilitation in COPD [29,40]. More specifically, patients graded MRC ≥3 are considered eligible for pulmonary rehabilitation services [9].

Rehabilitation facilitated by information and communication technologies is known as telerehabilitation [41,42]. Over the past few years, there has been an increase in telerehabilitation apps owing to the development of new technologies [43]. Compared with traditional inpatient or person-to-person rehabilitation, telerehabilitation allows for remote communication and is, therefore, more cost-effective for both health care providers and patients [43]. Some examples include in-home videoconferencing [44,45], mobile phone–based exercise programs [46], web-based self-monitoring [47-49], and more recently, socially assistive robots [50] and VR. Studies have suggested that telerehabilitation is suited to older people as it can improve the quality of life, allows for more independent living, is more convenient in terms of not having to travel for appointments, and enables cost-effective services [51-54]. However, despite growing evidence-based research demonstrating positive clinical outcomes and increasing telehealth utilization, patients are faced with equipment setup-related difficulties, limited scope of exercise, and connectivity [55].

Although the completion of pulmonary rehabilitation is associated with improved outcomes, including reduced subsequent hospital admission rates and better survival [10], the uptake and completion rates remain poor because of personal barriers associated with time, costs, fatigue, motivation, and inconvenience [8]. COPD is the third leading cause of death worldwide. In 2016, the Global Burden of Disease study reported a prevalence of 251 million global cases of COPD that year [56,57]. Research has indicated that COPD affects at least 320 million people and has a global economic cost of US $2.1 trillion [58,59]. Significant health care costs are associated with the treatment of exacerbations, including hospital visits and medication costs for maintenance therapy and outpatient treatment [24,29]. Over the past few years, general practice has been encouraged to explore new ways to improve COPD management and reduce hospital admissions [60]. A deeper understanding of the barriers associated with the acceptance and uptake of pulmonary rehabilitation by patients with COPD could reveal new methods to improve its uptake [10,60], which would reduce the considerable health care burden of COPD on the patients’ quality of life and the health economy. In addition, self-management interventions could lead to a reduction in COPD-related health costs by reducing hospital admissions [61-63]. Therefore, researchers and practitioners have sought innovative methods to create more exciting rehabilitation programs for patients such as VR, which is said to increase their excitement and interest, and excitement often leads to increased motivation to complete the rehabilitation program [21,64-66].

#### The Use of Virtual Reality for Physical Training and Rehabilitation

With recent technological advancements in VR, innovative approaches to improve traditional physical therapy and rehabilitation programs can be explored [21,67,68]. VR can be defined as a computer-assisted technology that provides the user with an immersive, interactive, and multisensory experience in a 3D virtual environment. The most cutting-edge hardware to facilitate VR is the head mounted display. Patients can control the digital recreations of their physical bodies (ie, avatars) to perform practice behaviors in the virtual environment [21]. Several studies have demonstrated that VR rehabilitation programs may develop physical outcomes [69-72]; outperform traditional rehabilitation programs [17,73,74]; and provide greater benefits to improve walking speed, balance, and mobility [75]. Many VR programs are designed with tasks that challenge the user, and well-designed VR rehabilitation programs could lead to improvements in cognitive, motor, and social aspects [76,77]. Given that VR can be accessed without supervision, an increased dosage of therapy can be provided without increasing staffing levels [77]. Remotely supervised VR interventions could complement conventional therapy programs and would be particularly suited in situations where cost savings are mandatory and/or when transport to the clinic is difficult for the patient [25]. From the patients’ perspective, VR environments for rehabilitation are naturally considered to be more exciting and enjoyable than traditional rehabilitation methods, which means that patients exert more effort and are more motivated to participate in VR rehabilitation compared with traditional rehabilitation methods [21,64,78,79]. As a result, patients benefit from greater autonomy and mobility in performing their daily activities [17]. However, few studies have investigated whether increased motivation explicitly leads to positive rehabilitation outcomes [21]. As VR is still a fairly recent addition to delivering physical therapy and rehabilitation, there is uncertainty on the benefits, despite the emerging research in this area [22,80]. However, as VR technology becomes more accessible and affordable, it will likely become more widely used in clinical rehabilitation settings [80-82]. Therefore, additional trials are required to evaluate the efficacy and determine the acceptability and feasibility of VR to guide future design and implementation of these systems in clinical practice [22,42].

## Methods

### Study Design

Patients participated in an 8-week trial using the Pulmonary Rehabilitation in Virtual Reality (*PR in VR*) program, and both qualitative and quantitative data were collected. During the trial, each patient was provided with a VR headset which was manufactured by Pico Interactive (Pico) Goblin and whose concept is developed by Concept Health Technologies. The headset was embedded with the PR in VR app and a small probe Nonin 3150 at home for an 8-week period. The patients wore the probe when exercising to measure their heart rate (HR) and oxygen saturation levels (displayed as a percentage). Pulse oximeter data were used by clinical staff to remotely measure the patients’ oxygen saturation and HR when the patients were exercising. The clinical staff used a web-based dashboard to observe these data remotely during the rehabilitation session. PR in VR was designed to enable patients to perform various exercises in their homes as displayed in the VR environment and comprised 8 separate modules. It was anticipated that the patients would spend at least 20 min per day using the VR rehabilitation program and complete 1 module per day during the 8-week period. Specifically, the app was divided into 2 subgroups: (1) education and (2) rehabilitation. Each subgroup has additional modules that can be accessed from within the app. The education section contains high definition videos with cinematic effects (visuals and audio) to increase retention of patients. The rehabilitation section (modules 5-7) comprises physical exercises led by a virtual instructor in the form of a 3D avatar. The physical exercises were drawn from the traditional pulmonary rehabilitation program and tailored to be suitable for patients while wearing the VR headset (eg, seated exercises). The final module (module 8) is a summary of the PR in VR program. The app is designed to send these data in real time to the server, and patients can monitor these data while exercising as they are displayed in the corner of the VR display screen.

### Participants

A total of 10 elderly patients with COPD (graded at MRC 4 or 5), aged between 63 and 75 years, were recruited in the United Kingdom. Overall, 6 patients from a health practice in South Cumbria and 4 patients from a general hospital in West Cumbria were recruited by the local physiotherapist at each location. Ethical approval was granted by the ethical committee of the Manchester Metropolitan University before data collection. Patients graded MRC 4 and 5 were provided the option to trial the PR in VR program or wait for 1 year to attend the traditional rehabilitation classes at the local rehabilitation center. All patients invited to take part in the trial agreed; however, because of the limited number of devices, only the patients who confirmed their interest first were recruited. There were 6 out of 10 male patients. Table 1 provides the patients’ demographic profile and entails the coding (patient [P]; P1-P10) used for the qualitative analysis. The average age of male patients in this study was 70 (SD 4.80) years, and the female participants were aged 3 years younger than the male participants on average (mean 67 years, SD 5.2 years).

**Table 1:** Demographic profile of the patients.

Patient number	Age (years)	Gender
P1	75	Male
P2	68	Male
P3	69	Male
P4	73	Female
P5	62	Female
P6	71	Male
P7	76	Male
P8	69	Female
P9	63	Female
P10	63	Male

### Data Collection

Before using PR in VR, the functional ability of the patients was assessed, and together with the physiotherapist, they were each asked to set a short-term goal (eg, increase walking distance without breathlessness) and a long-term goal (eg, feeling confident to leave the house on his/her own). The patients completed 5 self-reported surveys before and after using PR in VR.

In health and well-being research, qualitative methods are widely used as they provide insights into the perceptions and experiences of patients and health care professionals [83]. During June 2018, 2 focus groups and 6 one-to-one interviews were conducted in Cumbria. Each focus group was led by 2 researchers, 1 physiotherapist, and 1 health care assistant in each location. The first focus group, comprising 3 patients with COPD, was conducted at a health practice in South Cumbria and lasted approximately 75 min. Later, 4 one-to-one interviews were conducted by the physiotherapist in the same location and lasted between 11 min and 25 min each (interview 1: 12 min; interview 2: 11 min; interview 3: 15 min; and interview 4: 23 min). The second focus group, comprising 2 patients with COPD, took place at a general hospital in West Cumbria and lasted approximately 45 min. Then, the physiotherapist and health care assistant conducted 2 more one-to-one interviews at the same location, which lasted 16 min (interview 5) and 15 min (interview 6). The focus groups and interviews were conducted at the end of each patient’s 8-week VR-based rehabilitation program. During the focus groups and interviews, the questions aimed to explore how the patients benefited from the PR in VR program, explore how satisfied they were with using it, and identify the areas requiring further development. Drawing on the integrated educational aspect, the questions explored the effectiveness and benefits of immersive training in this specific context and further explored the usability of the VR device and app as well as the patients’ intention to use PR in VR in the future.

### Data Analysis

The qualitative data were transcribed verbatim before being analyzed using thematic analysis, which is a method used to identify patterns (ie, themes) within a dataset through a rigorous process of identification, analysis, organization, description, and reporting of those themes [84]. Thematic analysis is widely used in health care research [55,85-87] and was considered the most appropriate for this study as it is suited to questions related to people’s experiences or views and perceptions such as in this study.

In addition, the patients completed the 5 self-reported surveys before and after the trial to ensure that a comparison could be made between each patient’s scores. This was important to determine whether the patient showed improvements after using the PR in VR program. An analysis of the survey results before and after using VR was conducted using Excel.

## Results

### Qualitative Analysis

The purpose of the focus groups and interviews was to explore how the patients benefited from the PR in VR program, explore how satisfied they were with using it, and identify the areas requiring further development. In addition, the effectiveness and benefits of immersive learning in this specific context, the usability of the VR device and app, and the patients’ intention to use PR in VR in the future were explored. From the analysis, 11 themes emerged and are presented in Table 2. The themes are discussed in further detail below and provide support with direct quotations from the qualitative data collection.

**Table 2:** Themes and description of each theme.

Themes	Description
Increased compliance	Significant increase in the patients’ compliance with pulmonary rehabilitation (ie, doing their exercises)
Increased engagement	Increased engagement in pulmonary rehabilitation when using virtual reality because of enjoyment
Physical improvements	Significant improvements in patients’ physical health (ie, strength, mobility, and flexibility)
Improved psychological well-being	Patients’ psychological well-being has significantly improved
Improved health-related quality of life	Patients feel healthier and fitter (ie, they can confidently leave the house and socialize more than before)
Increased confidence	Significant improvements in confidence in terms of the patients managing their condition and performing daily activities and physical exercises
Patient satisfaction	Patients are satisfied with completing the Pulmonary Rehabilitation in Virtual Reality program and achieving their short-term and long-term goals
Increased feeling of security	Patients feel more secure, reassured, and confident to exercise, knowing their physiological data are being remotely supervised
Effective immersive learning	Effectiveness of immersive learning for patients with COPD^a^ was demonstrated
Personalized programs	A recommendation to provide programs tailored to suit various levels of COPD
Need for technological improvements	Need for technological improvements of both the device (eg, a more lightweight headset) and the content of the app (eg, additional functions to control the pace and standing-up exercises) to improve the patients’ experience

^a^COPD: chronic obstructive pulmonary disease.

### Increased Compliance

The findings demonstrate that PR in VR significantly increased the patients’ (P1-P10) compliance with the frequency and consistency of performing their exercises. For example, patients had difficulty attending the rehabilitation center because of barriers associated with time and other personal commitments taking priority:

I couldn’t complete traditional rehabilitation. There was always something cropped up.

P9

In comparison, using PR in VR remotely was “easier” (P9) and “pleasurable rather than chore-like” (P9), given that it could be integrated into their daily routine in a more flexible basis. For patients with COPD, this is an important factor as several patients (P1-P10) mentioned that some days they do not feel well enough, physically and/or mentally, to travel to the rehabilitation center. Therefore, being able to access pulmonary rehabilitation anywhere at any time is highly beneficial and attractive for this target group:

VR is more akin to my needs. I did not feel like traditional classes were doing anything for me.

P9

I prefer to do it at home, partly because of getting to the venue.

P2

### Increased Engagement

Compared with previous pulmonary rehabilitation methods, PR in VR increased engagement for all participants (P1-P10) as it was more enjoyable, and more importantly, having the 3D avatar gave the experience a social aspect. Therefore, patients were more disciplined with PR in VR than they had been with previous methods such as the booklet:

I was amazed how I actually looked forward to doing [PR in VR] and getting on it and seeing the chap and doing [the exercises] with him.

P8

You discipline yourself to use it. If it’s not there, then I don’t think I would. You just don’t do them [exercises using the booklet]...but [with VR] the guy is there and he’s talking to you, and you feel as though you are with the two people, it’s just the feeling it gives you.

P9

Patients were more engaged with PR in VR as they felt more comfortable performing their exercises in their home environment at their own leisure:

Because it was at home, I think I did it more. Whereas I would have been ringing the class to tell them I cannot make it because I don’t feel well enough.

P8

### Physical Improvements

Participants (P1, P3-P5, and P7-P10) demonstrated physical improvements, including increased strength, mobility, and flexibility:

I know certainly my legs are stronger. I can feel that even just getting up off the chair.

P8

I can’t walk to the car without having to stop usually. But when I am not doing the exercises, I could walk to the car without having to stop.

P4

I am a lot more flexible and without the pain!

P1

Several exercises in the PR in VR program focused on developing strength in the thigh muscles, which subsequently improved the patients’ cardiovascular fitness, enabling them to walk further (P1-P10). In addition, patients reported increased strength in their upper body, which resulted in them feeling less breathless and having a much quicker recovery time when breathlessness occurred*.* As a result of the consistent physical activity, as opposed to minimal daily movement, patients felt healthier both physically and mentally:

After using VR, I am less breathless...my recovery time is much quicker.

P9

I do feel healthier in myself...I feel a lot better with myself, with the movement, physically. I used to just sit in my chair and do nothing.

P1

### Improved Psychological Well-Being

In relation to the previous theme (physical improvements), the patients’ psychological well-being significantly improved:

When I’ve finished the Thai Chi, I do feel physically and mentally relieved.

P1

All patients (P1-P10) confirmed that they would recommend the PR in VR program to others in their situation largely because of the psychological benefits, which are particularly important for patients with COPD, given the associated mental health problems (eg, depression) often experienced from the initial diagnosis to adapting to living with COPD. In line with this, motivation played a key role in the patients’ enthusiasm to continue with PR in VR. Overall, this could assist in overcoming mental health problems and improving their general psychological well-being:

Motivation is my main reason to continue. Because I get depressed really easily.

P4

It gets me motivated and I’m in the [wheel]chair a lot because of my health so doing the exercises does motivate me but sometimes I don’t like being honest about it, but I have to mentally build up to do it because of the way I am feeling. But yes, I would recommend it.

P3

### Improved Health-Related Quality of Life

Improvements in physical and psychological well-being were positively associated with health-related quality of life (HRQoL), as demonstrated by all participants (P1-P10). Performing daily activities was more enjoyable than before, and patients were more sociable:

It has made my daily activities easier...I am more comfortable with what I do.

P9

I am getting out and about now.

P7

For example, P4 had not left the house for almost 2 months before starting PR in VR. In addition, patients felt happier as they could spend more quality time with friends and family:

From sitting on a sofa, I had been able to go with my husband for a coffee, to go out in the car, and I had not done any of that for 7 weeks. It had got me to the stage again where I felt I could go out and go shopping with the trolley. I have done none of that for about 2 months.

P4

Going out every day has made a difference; I have been seeing my friends.

P7

### Increased Confidence

Most patients (P1-P5 and P7-P10) reported significant improvements in confidence in terms of managing their condition, conducting daily activities, participating in social activities, and managing their breathing:

I can get upstairs in one go now.

P7

It builds my confidence up because I can go out more.

P4

It has given me more confidence in breathing along with what I am doing.

P9

Reflecting on this, patients discussed how they had previously lost motivation and confidence in their ability to manage tasks themselves without the support of their partners and several had “given up” (P3, P4, and P7-P10). However, with the PR in VR program, the patients had gained back more control in their lives:

I think I had given up. I decided that the illness had gone so far that I was not going to get better, I was not going to get out again, or enjoy life. It has completely reassured me that way and given me the energy to get going again.

P8

Possibly it was a mental thing. I felt confident enough. I started filling my own flasks.

P7

### Patient Satisfaction

Successful completion of their exercises within a given day using PR in VR was positively associated with patient satisfaction (P1-P10). Noticing improvements in physical wellness enabled patients to feel satisfied and confirmed the benefits associated with following the PR in VR program:

What I enjoyed most was knowing that I could get through the exercises. Knowing that my oxygen was not dropping. It was a real achievement for me. I was used to going to the toilet and coming back and having to catch my breath. All of a sudden, I could do these exercises and after a few times I was still breathing normal.

P8

Given that PR in VR is self-managed and remotely supervised, patients’ self-motivation played a critical factor in their consistency with exercising and subsequent self-satisfaction:

I feel satisfied that I motivated myself to do it. It did help me to do it. I feel satisfied when I sit down and think that’s me done for tonight.

P4

### Increased Feeling of Security

A primary concern for patients was experiencing exasperation when exercising alone at home. In comparison to previous methods, PR in VR is advantageous as it allows for remote supervision, meaning that the patients’ physiological data (ie, HR and oxygen level) are continuously tracked and monitored by health practitioners. Therefore, this function improved the patients’ experience as they felt more secure (P9), confident (P7 and P9), and reassured (P8), knowing that they were being monitored while exercising in real time should exacerbation occur:

The minute I am getting out of breath [at home] I go into panic.

P8

It has given me more confidence in breathing along with what I am doing.

P9

It reassured me being able to see my oxygen level and heart rate while I was exercising. I feel it has increased my confidence.

P8

Furthermore, this function assisted patients with better management of their condition and to avoid overexerting themselves into exasperation:

[PR in VR] has improved my breath...in the VR it shows the oxygen go up and back down again, it gave me that back again where I could settle myself down.

P8

### Effective Immersive Learning

PR in VR provides patients with information on COPD with the aim of helping them better manage their condition. The findings demonstrated the effectiveness of immersive learning for patients with COPD as it educated them on the disease, which some participants were never informed of. For instance, P1 found the learning aspect useful and “very informative to find out what is actually happening.” During the focus groups, P1 could reiterate what he had learned from the program 5 weeks prior:

That’s the first time I have seen on screen what emphysema actually is.

P1

The PR in VR program had increased the patients’ awareness of their breathing techniques (P1, P3, and P8) and the variety of exercises required to target various muscles and support their overall health and well-being. The findings further support the effectiveness of immersive learning, specifically using VR for patients with COPD:

He says breath out when you do the exertion, I was not doing that before...it has improved my breath...the voice over was helpful and reassuring.

P8

I think it’s a good idea. It reminds you that you’ve got to do different exercises to target different muscles.

P3

### Personalized Programs

Recommendations to improve the patients’ experience of PR in VR were provided. Patients (P1-P6, P8, and P10) suggested various levels of exercises (ie, harder set of exercises and easier set of exercises) to suit the many levels of patients with COPD to improve the program. More specifically, patients (P2, P5, and P10) explained how the first 2 to 3 levels were “too easy” (P5), slow paced (P5), and too long (P10) and were eager to push onto more “severe” modules (P10)*.* Therefore, these patients required more advanced exercises to ensure that they were constantly being challenged. On the contrary, the more advanced exercises were too challenging for other patients (P1, P3, P4, and P5) on the first attempt and required repetition, given that they might not have exercised for a prolonged period before using PR in VR. Indeed, those patients enjoyed the flexibility of being able to move back and forth throughout the modules depending on their well-being at that time:

It is beneficial to have the different levels so I can drop down depending how I feel each day.

P4

Furthermore, it was suggested that a variety of exercises at the same difficulty level would retain engagement:

I would like two or more routines in each module instead of doing the same thing all the time. For longer than six weeks it would become boring.

P5

### Needs for Technological Improvements

Furthermore, to improve the patients’ experience of PR in VR, technological improvements of both the device and app were required (P1-P10). For instance, the main technical issue within the app whereby the camera moved to the right and took several seconds to re-center was a recurrent topic of conversation*.* Hence, improved graphics could enhance the experience but was considered a minor factor in the overall rehabilitation experience that could easily be solved:

The whole thing was excellent it really was except for the glitches but I am sure that can be overcome.

P5

Suggestions for additional functions included a fast-forward and pause button (P1-P5) to allow patients to have more control over the program and pause it while they recovered:

If you could pause it when you know that you’re going to get out of breath.

P1

All patients (P1-P10) found the headset easy to use; however, some required it to be more lightweight (P4, P5, and P9). With regard to the content, some patients (P1-P5) enjoyed the standing-up exercises.

### Quantitative Data Analysis

In addition to the focus groups and interviews, quantitative data were collected, and the findings further support the qualitative findings. Table 3 presents the results of the outcome measures. The average of each of the 5 surveys was calculated based on the patient data gathered before and after the VR rehabilitation program. As can be seen, the findings demonstrate an improvement of all outcome measures after patients used the VR program. The Chronic Respiratory Disease Questionnaire (CRQ) measures both physical and emotional aspects of chronic respiratory disease. The results indicate that the patients had improved dyspnea, fatigue, and emotional function after completing VR-assisted treatment. The female participants collectively showed more improvements in both dyspnea and emotional function than the male participants. The Patient Health Questionnaire-9 and generalized anxiety disorder-7 demonstrated that a substantial proportion of patients had reduced feelings of depression and anxiety, respectively, which further supported the theme of improvements in the patients’ psychological well-being. The Short Physical Performance Battery is used to assess lower extremity physical performance status by combining the results of the gait speed, chair stand, and balance tests. The results of the Edmonton Frail Scale and Short Physical Performance Battery were positively associated with the patients’ physical improvements, including strength, mobility, and flexibility. Finally, the Patient Activation Measure results demonstrated the patients’ progression with knowledge, skills, and confidence in self-managing their condition after using VR.

**Table 3:** Outcome measure results before and after using virtual reality.

Outcome measures	Before using virtual reality, mean (SD)	After using virtual reality, mean (SD)
Short Physical Performance Battery	6.78 (2.95)	8.43 (1.72)
CRQ^a^–dyspnea	2.22 (1.09)	2.96 (1.15)
CRQ–fatigue	3.11 (1.43)	3.27 (1.15)
CRQ–emotional	3.85 (1.52)	4.36 (1.01)
CRQ–mastery	3.83 (1.19)	4.22 (0.74)
Patient Activation Measure	60.81 (10.83)	63.46 (15.89)
Edmonton Frail Scale	6.56 (2.07)	5.38 (2.00)
Patient Health Questionnaire-9	8.44 (5.90)	6.11 (4.76)
Generalized Anxiety Disorder-7	5.78 (5.89)	4.22 (3.11)

^a^CRQ: Chronic Respiratory Disease Questionnaire.

## Discussion

### Principal Findings

This study aimed to assess the benefits and outcomes of VR as an innovative method for pulmonary rehabilitation in 2 aspects. First, the study aimed to investigate whether VR improves compliance with pulmonary rehabilitation in patients with COPD, particularly the vulnerable patient group (MRC 4 or 5). Finding innovative pulmonary rehabilitation treatment methods that engage this particular patient group is vital, given the severity of the disease that affects the HRQoL and the barriers associated with attending traditional rehabilitation classes [12-15]. This study demonstrates that PR in VR significantly increases the patients’ compliance with pulmonary rehabilitation compared with traditional methods mainly because of the flexibility to exercise at any time of the day and location and as it is not restricted to home- or clinic-based scenarios, which is more accommodating for their daily psychological and physical well-being [88]. The second research question was to investigate whether VR provides a credible alternative to traditional pulmonary rehabilitation. Thematic analysis revealed nine themes illustrating the benefits associated with PR in VR compared with traditional methods.

### Conclusions

The patients demonstrated significant improvements in physical ability and psychological well-being because of their consistency with exercises, thus improving their HRQoL. These findings are consistent with both the quantitative results of this study and previous research investigating the benefits of VR for other chronic diseases [17,64,69-71,73,74,78] and provide evidence specific to patients with COPD. Owing to its remote accessibility, PR in VR provides a solution to overcome the associated barriers of attending traditional rehabilitation classes (ie, transport and travel, fatigue, motivation, inconvenience, burden of illness, disruption caused to daily activities, low awareness of rehabilitation, and knowledge of and disbelief in the beneficial outcomes of the rehabilitation) [12-15]. Indeed, patients feel secure and confident enough to exercise without face-to-face supervision as health practitioners can remotely monitor their physiological performance data when using PR in VR. Pulmonary rehabilitation is delivered to a low proportion of the population despite its demonstrated benefits for patients with COPD [8]. However, PR in VR can be deployed at scale, which provides a solution to the recurring issue of too long a waiting list for rehabilitation classes [10] and allows health practitioners to measure patient performance in real-time. Overall, this study demonstrates how remotely supervised VR-based pulmonary rehabilitation can help to overcome current issues and limitations associated with providing this service to patients with COPD at scale. Compared with previous methods, VR could provide a more cost-effective solution for the health treatment to deliver pulmonary rehabilitation to a vast majority of this patient group nationwide.

### Contribution, Implications, and Future Research

#### Key Contributions

Previous studies have investigated the benefits of using VR for rehabilitation for long-term conditions [17,18,89]. However, its benefits for self-managed and remotely supervised pulmonary rehabilitation remain unexplored, and to the best of the researchers’ knowledge, this study is one of the first to provide empirical evidence using qualitative data that are further supported by quantitative results. Therefore, this study contributes important findings demonstrating that PR in VR represents an effective form of self-managed and remotely supervised pulmonary rehabilitation that can be delivered at scale. Overall, this study provides a significant contribution to health care research investigating the benefits of implementing digital technologies to improve rehabilitation, with particular contribution to pulmonary rehabilitation research. Moreover, this study provides important practical implications for the health care industry and medical practitioners on the benefits of VR for pulmonary rehabilitation. PR in VR is an innovative app distinct from previous self-managed rehabilitation methods as it allows health practitioners to supervise patients with COPD remotely and at scale and to immediately measure patient performance. This investigation offers proof of concept that PR in VR intervention can be used for elderly patients with COPD. With evidence, this alternative intervention platform provides an interactive and visually stimulating approach for use in clinical treatment and could provide pulmonary rehabilitation to patients with COPD who cannot easily access traditional rehabilitation methods.

#### Possible Future Research

This study has several limitations that could be mitigated by further research. The first limitation pertains to the limited sample size; however, this is common in other exploratory studies investigating VR for rehabilitation [18,89]. This study employed 10 patients with COPD, aged between 63 and 75 years, from the United Kingdom. Although small sample sizes can provide important initial inferences about a topic, they can provide few firm conclusions [21]. However, as this is the first study investigating VR for pulmonary rehabilitation, a small sample size offers promising pilot data as a first phase trial. Furthermore, COPD in the United Kingdom is common among people aged ≥40 years [90]; therefore, it is important that future studies employ a larger sample covering the majority of the respective age groups.

## Abbreviations

COPD: chronic obstructive pulmonary disease
HR: heart rate
HRQoL: health-related quality of life
MRC: Medical Research Council
PR: pulmonary rehabilitation
VR: virtual reality
